# Positive RT-PCR Test Results in 420 Patients Recovered From COVID-19 in Wuhan: An Observational Study

**DOI:** 10.3389/fphar.2020.549117

**Published:** 2020-10-07

**Authors:** Shaobin He, Jiaxing Tian, Xiaodong Li, Yana Zhou, Mingzhong Xiao, Ying Zhang, Xiaojun Min, Xiuyang Li, De Jin, Qing Zhang, Yujiao Zheng, Jia Ke, Qingwei Li, Junxiu Tao, Ping Song, Han Wang, Yi Lv, Qiyou Ding, Shuang Tang, Jiaran Lin, Zhaoyuan Jiang, Zijun Zhang, Juexian Song, Fengmei Lian, Xiaolin Tong

**Affiliations:** ^1^ Hubei Hospital of Traditional Chinese Medicine, Wuhan, China; ^2^ Hubei University of Chinese Medicine, Wuhan, Wuhan, China; ^3^ Departments of Endocrinology, Guang’anmen Hospital, China Academy of Chinese Medical Sciences, Beijing, China; ^4^ Liver Disease Branch, Hubei Hospital of Traditional Chinese Medicine, Wuhan, China; ^5^ Department of Oncology, Hubei Hospital of Traditional Chinese Medicine, Wuhan, China; ^6^ Center for Evidence-based Chinese Medicine, Beijing University of Chinese Medicine, Beijing, China; ^7^ Department of Endocrinology, Hubei Hospital of Traditional Chinese Medicine, Wuhan, China; ^8^ Medical Affairs Department, Guang’anmen Hospital, China Academy of Chinese Medical Sciences, Beijing, China; ^9^ Department of Orthopedics, Hubei Hospital of Traditional Chinese Medicine, Wuhan, China; ^10^ Graduate School, Beijing University of Chinese Medicine, Beijing, China; ^11^ Department of Pulmonary Medicine, Hubei Hospital of Traditional Chinese Medicine, Wuhan, China; ^12^ International Cooperation Department, China Academy of Chinese Medical Sciences, Beijing, China; ^13^ Department of Neurology, Xuanwu Hospital, Capital Medical University, Beijing, China

**Keywords:** coronavirus disease 2019, comprehensive intervention, correlation factor analysis, recurrence rate, positive RT-PCR test result

## Abstract

**Objective:**

During the follow-up of patients recovered from coronavirus disease 2019 (COVID-19) in the quarantine and observation period, some of the cured patients showed positive results again. The recurrent positive RT-PCR test results drew widespread concern. We observed a certain number of cured COVID-19 patients with positive RT-PCR test results and try to analyze the factors that caused the phenomenon.

**Methods:**

We conducted an observational study in COVID-19 patients discharged from 6 rehabilitation stations in Wuhan, China. All observed subjects met the criteria for hospital discharge and were in quarantine. Data regarding age, sex, body mass index (BMI), course of disease, comorbidity, smoking status and alcohol consumption, symptoms in and out of quarantine, and intervention were collected from the subjects’ medical records and descriptively analyzed. The main outcome of this study was the RT-PCR test result of the observed subjects at the end of quarantine (negative or positive). Logistic regression analysis was used to identify the influencing factors related to recurrent positive RT-PCR test results.

**Results:**

In this observational study, 420 observed subjects recovered from COVID-19 were included. The median age was 56 years, 63.6% of the subjects were above 50 years old, and 50.7% (213/420) were female. The most common comorbidities were hypertension [26.4% (111/420)], hyperlipidemia [10.7% (45/420)], and diabetes [10.5% (44/420)]. 54.8% (230/420) manifested one or more symptoms at the beginning of the observation period, the most common symptoms were cough [27.6% (116/420)], shortness of breath 23.8% (100/420)], and fatigue [16.2% (68/420)], with fever rare [2.6% (11/420)]. A total of 325 subjects were exposed to comprehensive intervention; 95 subjects were absence of intervention. The recurrence rate of positive RT-PCR test results with comprehensive intervention was 2.8% (9/325), and that with no intervention was 15.8% (15/95). The results of logistic regression analysis showed that after adjusted for factors such as age, sex, and comorbidity and found out that comprehensive intervention was correlated with the recurrent positive RT-PCR test results. There was appreciably less recurrence in the comprehensive intervention group.

**Conclusions:**

The factors related to positive RT-PCR test results in observed subjects recovered from COVID-19 were age, comorbidity, and comprehensive intervention, among which comprehensive intervention might be a protective factor.

**Clinical Trial Registration:**

Chictr.org.cn, identifier ChiCTR2000030747.

## Introduction

By March 11^th^, 2020, 121,133 cases were diagnosed as coronavirus disease 2019 (COVID-19) globally. In China, 80,967 cases have been diagnosed, among which 61661 have been cured and discharged from the hospital (Chinese Center for Disease Control and Prevention). In view of sequelae in cured patients with severe acute respiratory syndrome (SARS), numerous discharged patients have drawn public attention. Recently, it was reported that some COVID-19 patients who had met the criteria for hospital discharge (absence of clinical symptoms and radiological abnormalities with 2 consecutive negative RT-PCR test results) showed positive RT-PCR test results for COVID-19 nucleic acid later (Lan et al., 2020). The patients usually had no or mild clinical symptoms; however, their health status and infectivity were unclear, which caused widespread concern to the key points which affected the control of the disease, including the complexity of COVID-19, discharge criteria, reinfection after discharge, infectivity of discharged patients with positive RT-PCR test results, quality of nucleic acid kit and specimen sampling, and obstructed to epidemic prevention and control. Currently, most researchers focus on the epidemiological characteristics of COVID-19 patients, as well as the clinical manifestations and efficacy outcomes. However, few studies have been conducted on patients who have recovered and been discharged, which has significantly affected our complete understanding of the disease. In Wuhan, with the implementation of 14-day quarantine measures for discharged COVID-19 patients, we observed a certain number of cured COVID-19 patients with RT-PCR test results in and out of the quarantine and tried to analyze the factors that caused this phenomenon. The study was approved by the Medical Ethics Committee of Hubei Provincial Hospital of Traditional Chinese Medicine (no. HBZY2020-C01-01).

## Methods

### Study Design and Participants

We conducted an observational study using data from six rehabilitation stations: Wuhan Vocational College of Software and Engineering (WVCSE) rehabilitation station, the City Economic Hotel on Chunghwa Road, Galaxy Kindom Hotel on Yangyuan Street, Lavande Hotel on Jiajiashan Street, You Melody Hotel on Liangdao Street, and Home Inn on Liangdao Street. All the COVID-19 patients observed in this study had been hospitalized and discharged before, so they were all tested negative for RT-PCR when included. The current COVID-19 discharge criteria are as follows: 1) body temperature is back to normal for more than 3 days; 2) respiratory symptoms improve obviously; 3) pulmonary imaging shows obvious absorption of inflammation, 4) nucleic acid tests negative twice consecutively on respiratory tract samples such as sputum and nasopharyngeal swabs (sampling interval being at least 24 h). All observed subjects met the above discharge criteria and were in quarantine. If the RT-PCR test was still negative after 14 days from discharge, then they can be released from quarantine. During the observed period, some patients were administered comprehensive intervention, and some were not. The comprehensive interventions included: (1) Baduanjin exercise (Zhao et al., 2019), was taught by a professional instructor combined with recorded videos. The exercise time was 15 min per day during 10:00–10:15 in the morning or 15:00–15:15 in the afternoon. (2) Foot baths (Vyas et al., 2019) were performed 1 h before bedtime for 20 min daily. The temperature of water in the foot bath should be controlled at 38–40°C, and people with skin ulcers on their feet should not undergo this therapy. (3) Moxibustion with acupoint application (Shou et al., 2020), which was a Type II acupoint plaster for intervening cough from Wuhan Guojiu Technology Development Co., Ltd. (Registration no. Hubei Drug Administration Machinery (Zhun) Zi 2002 no. 2260633); the selected acupoints included CV22 and GV14. The instructions were to apply 1 paste of the Type II Acupoint plaster for intervening cough on acupoints CV22 and GV14, once a day for 12 h. Pregnant women and patients with diabetes, skin allergies, skin ulceration, and acute contusion bleeding disorders were prohibited from using this therapy. (4) Tongzhi Granule, administered to 1 bag (dissolved in 200 mL of water at 95°C) per day, 30 min after breakfast, and 30 min after dinner. (5) Wuhan Kangyi Decoction, administered to 2 bags (dissolved in 200 mL of water at 95°C) 30 min after breakfast and dinner, respectively. The above therapies can be chosen and combined based on individual symptoms. Considering that we need to evaluate the recurrent rate of positive RT-PCR test results of the population, we excluded the suspected cases and clinically diagnosed cases of COVID-19; convalescents in quarantine with RT-PCR testing were included. In the study, subjects were divided into the comprehensive intervention and the no intervention.

### Procedures

The results of this study were analyzed and reported in accordance with the STROBE guidelines. Prior to January 23, 2020, laboratory confirmation of SARS-CoV-2 was performed at the Chinese Center for Disease Control and Prevention (CDC); subsequently, laboratory confirmation was performed at certified tertiary hospitals. The RT-PCR test was based on the criteria provided by the World Health Organization (WHO) (World Health Organization, 2020). We obtained medical records of diagnosed COVID-19 patients who were discharged from the hospital and were in quarantine from February 22, 2020 to March 10, 2020 (National Health Commission of the People’s Republic of China, 2020a). The nucleic acid kit (fluorescent RT-PCR) was recommended by the CDC, and extraction of nucleic acid from clinical samples (including uninfected cultures that served as negative controls) was performed as the description of the manufacturer (BGI Biotechnology Co., Ltd). Data on demographic and clinical characteristics, comorbidity, course of disease, smoking status, and alcohol consumption were extracted. Symptoms of the observed subjects in and out of the rehabilitation station and comprehensive intervention were also recorded. If the relevant information was missing, we directly contacted the patient’s family. Data for the study were collected and examined manually by two researchers, and differences were resolved through consultation by a third researcher.

### Outcomes

The primary outcome of this study was the RT-PCR test result of the observed subjects at the end of quarantine (negative or positive).

In addition, we performed a descriptive analysis of the demographic characteristics. Age, sex, body mass index (BMI), course of disease, comorbidity, smoking status and alcohol consumption, symptoms in and out of quarantine, and intervention were descriptively analyzed. We also compared the patients according to different interventions, and each intervention was considered a factor. Logistic regression analysis was used to compare positive RT-PCR test results in convalescents with intervention, age, sex, BMI, course of disease, comorbidity, smoking status, and alcohol consumption and to identify the influencing factors related to recurrent positive RT-PCR test results.

### Statistical Analysis

Numerical variables were summarized as mean (± SD) if the data are normally distributed or median variables were presented (interquartile range, IQR) if they are not. The data of the categorical variables were described as counts and percentages. The characteristics of the subjects and the different interventions (comprehensive invention and no intervention) were described. The characteristic variables included age, sex, BMI, comorbidity, course of disease, smoking status, alcohol consumption, and symptoms in and out of quarantine. Univariate analysis was used to analyze the characteristics of different interventions. Multivariable logistic regression was used to analyze possible independent factors that influence recurrent positive RT-PCR test results. *P*-values < 0.05 were considered statistically significant. The OR value and 95%CI were used to estimate the effect size. Statistical analyses were performed using SPSS 19.0 software (SPSS Inc., Chicago, IL, United States).

## Results

**Figure 1:**
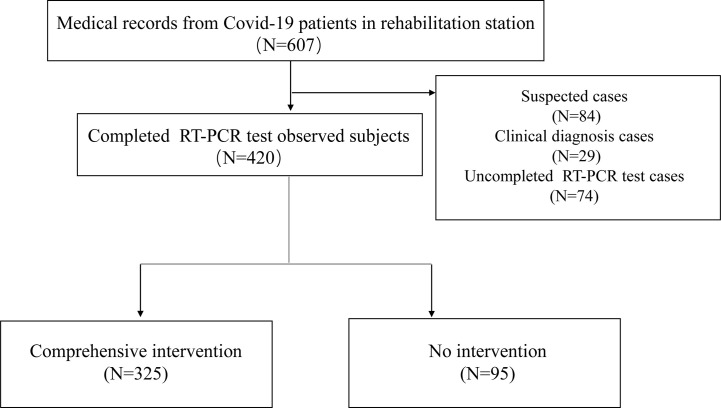
Flow chart of the observational research.

By March 10, 2020, the data of 607 cases from 6 rehabilitation stations in Wuhan was collected, including 84 suspected cases, 29 clinically diagnosed cases and 494 former diagnosed cases. 420 former diagnosed patients have completed the RT-PCR testing and were included in the study, among which 325 observed subjects were administered comprehensive intervention, 95 subjects didn’t receive any intervention (**Figure 1**).

### Demographic and Clinical Characteristics

In 420 observed subjects, the median age was 56 years, 63.6% of the subjects were above 50 years old, 50.7% (213/420; 95% CI: 45.9–55.5%) were female. 52.2% (219/420; 95% CI:47.4–56.9%) of the subjects were overweight or obese (BMI≥24), and 41.0% (172/420; 95% CI: 36.2–45.7%) had one or more comorbidities. The most common comorbidities were hypertension [26.4% (111/420; 95% CI: 22.2–30.6%)], hyperlipidemia [10.7% (45/420; 95% CI:7.8–13.7%)] and diabetes [10.5% (44/420; 95% CI: 7.5–13.4%)]. 54.8% (230/420; 95% CI: 50–59.5%) manifested one or more symptoms at the beginning of the observation period, the most common symptoms were cough [27.6% (116/420; 95% CI: 23.3–31.9%)], shortness of breath 23.8% (100/420; 95% CI: 19.7–27.9%)] and fatigue [16.2% (68/420; 95% CI:12.7–19.7%)], with fever rare [2.6% (11/420; 95% CI:1.1–4.1%)]. 10.5% (44/420; 95% CI:7.5–13.4%) of the subjects were smokers and 10.5% (44/420; 95% CI: 7.5–13.4%) with alcohol consumption. The duration of disease was defined as time from onset to the time of RT-PCR testing. The subjects’ median course of disease was 40 days.

Between 325 subjects with comprehensive intervention and 95 subjects without intervention, there were differences in the age (54 vs. 58), comorbidity (36.6 vs. 55.8%), and symptom (51.4 vs. 66.3%) at the beginning of the observation period. Subjects in the non-intervention group had more comorbidities and symptoms at the beginning of the observation period and were older. While there was no significant difference between the two groups in terms of sex, BMI, course of disease, smoking status, and alcohol consumption (**Table 1**).

**Table 1:** Characteristics of study observed person.

Characteristic		All patients	Comprehensive intervention	No intervention
**Age**				
Median (IQR)-year		56(43–63.75)	54(42–62)	58(48–68)
Distribution-no./total no. (%; 95% CI)				
	0–14 years	1/420(0.2; -0.2–0.7)	0/325(0)	1/95(1.1; -1.0–3.1)
	15–49 years	152/420(36.2;31.6–40.8)	126/325(38.8;33.5–44.1)	26/95(27.4; 18.4–36.3)
	50–64 years	172/420(41.0;36.2–45.7)	139/325(42.8;37.4–48.1)	33/95(34.7; 25.2–44.3)
	≥65 yeats	95/420(22.6;18.6–26.6)	60/325(18.5;14.2–22.7)	35/95(36.8; 27.1–46.5)
**Sex**				
	Female sex-no./total no. (%)	213/420(50.7;45.9–55.5)	160/325(49.2;43.8–54.7)	53/95(55.8; 45.8–65.8)
**Body mass index**				
Median (IQR)-kg/m^2^		24.05(21.99–26.02)	24.06(21.97–26.18)	24.03(22.49–25.48)
Distribution-no./total no. (%; 95% CI)				
	<18.5kg/m^2^	12/420(2.9;1.3–4.5)	10/325(3.1;1.2–0.5)	2/95(2.1; -0.8–5)
	18.5 ≤ BMI<24kg/m^2^	189/420(45.0;40.2–49.8)	145/325(44.6;39.2–50)	44/95(46.3; 36.3–56.3)
	24≤BMI<27kg/m^2^	146/420(34.8;30.2–39.3)	111/325(34.2;29–39.3)	35/95(36.8; 27.1–46.5)
	BMI≥27kg/m^2^	73/420(17.4;13.8–21)	59/325(18.2;14–22.3)	14/95(14.7; 7.6–21.9)
**Course of disease**				
	Median (IQR)-d	40(33–43)	39(33–43)	40(35–44)
**Symptoms during observation period -no./total no. (%; 95% CI)**				
	Total	230/420(54.8; 50–59.5)	167/325(51.4; 46.0–56.8)	63/95(66.3; 56.8–75.8)
	Cough	116/420(27.6; 23.3–31.9)	82/325(25.2; 20.5–30.0)	34/95(35.8; 21.6–45.4)
	Shortness of breath	100/420(23.8; 19.7–27.9)	75/325(23.1; 18.5–27.7)	25/95(26.3; 17.5–35.2)
	Fatigue	68/420(16.2; 12.7–19.7)	54/325(16.6; 12.6–20.7)	14/95(14.7; 7.6–21.9)
	Insomnia	37/420(8.8; 6.1–11.5)	8/325(2.5; 0.8–4.1)	29/95(30.5; 21.3–39.8)
	Inappetence	36/420(8.6; 5.9–11.2)	26/325(8.0; 5.1–10.9)	10/95(10.5; 4.4–16.7)
	Sweating	30/420(7.1; 4.7–9.6)	12/325(3.7; 1.6–5.7)	18/95(18.9; 11.1–26.8)
	Diarrhea	29/420(6.9; 4.5–9.3)	22/325(6.8; 4–9.5)	7/95(7.4; 2.1–12.6)
	Limb pain	22/420(5.2; 3.1–7.4)	22/325(6.8; 4–9.5)	–
	Thirsty	18/420(4.3; 2.3–6.2)	1/325(0.3; -0.3–0.9)	17/95(17.9; 10.2–25.6)
	Nausea and vomiting	15/420(3.6; 1.8–5.3)	15/325(4.6; 2.3–6.9)	–
	Fever	11/420(2.6; 1.1–4.1)	11/325(3.4; 1.4–5.4)	0/95(0)
	Constipation	9/420(2.1; 0.8–3.5)	–	9/95(9.5; 3.6–15.4)
	Fear of wind	3/420(0.7; -0.1–1.5)	2/325(0.6; -0.2–1.5)	1/95(1.1; -1.0–3.1)
**Symptoms after observation period -no./total no. (%; 95% CI)**				
	Total	241/420(57.4; 52.7–62.1)	173/325(53.2; 47.8–58.7)	68/95(71.6; 62.5–80.6)
	Insomnia	95/420(22.6; 18.6–26.6)	63/325(19.4; 15.1–23.7)	32/95(33.7; 24.2–43.2)
	Cough	94/420(22.4; 18.4–26.4)	63/325(19.4; 15.1–23.7)	31/95(32.6; 23.2–42.1)
	Shortness of breath	88/420(21.0; 17.1–24.8)	55/325(16.9; 12.8–21.0)	33/95(34.7; 25.2–44.3)
	Sweating	69/420(16.4; 12.9–20)	53/325(16.3; 12.3–20.3)	16/95(16.8; 9.3–24.4)
	Expectoration	55/420(13.1; 9.9–16.3)	39/325(12.0; 8.5–15.5)	16/95(16.8; 9.3–24.4)
	Thirsty	52/420(12.4; 9.2–15.5)	29/325(8.9; 5.8–12)	23/95(24.2; 15.6–32.8)
	Fatigue	38/420(9.0; 6.3–11.8)	17/325(5.2; 2.8–7.7)	21/95(22.1; 13.8–30.4)
	Diarrhea	26/420(6.2; 3.9–8.5)	16/325(4.9; 2.6–7.3)	10/95(10.5; 4.4–16.7)
	Inappetence	18/420(4.3; 2.3–6.2)	10/325(3.1; 1.2–5.0)	8/95(8.4; 2.8–14.0)
	Limb pain	17/420(4.0; 2.2–5.9)	17/325(5.2; 2.8–7.7)	–
	Constipation	16/420(3.8; 2.0–5.6)	5/325(1.5; 0.2–2.9)	11/95(11.6; 5.1–18)
	Fever	9/420(2.1; 0.8–3.5)	3/325(0.9; -0.1–2.0)	6/95(6.3; 1.4–11.2)
**Comorbidity**				
	Total	172/420(41.0; 36.2–45.7)	119/325(36.6; 31.4–41.9)	53/95(55.8; 45.8–65.8)
	Hypertension	111/420(26.4; 22.2–30.6)	87/325(26.8; 22–31.6)	24/95(25.3; 16.5–34)
	Hyperlipidemia	45/420(10.7; 7.8–13.7)	35/325(10.8; 7.4–14.1)	10/95(10.5; 4.4–16.7)
	Diabetes	44/420(10.5; 7.5–13.4)	31/325(9.5; 6.3–12.7)	13/95(13.7; 6.8–20.6)
	Coronary heart disease	23/420(5.5; 3.3–7.7)	16/325(4.9; 2.6–7.3)	7/95(7.4; 2.1–12.6)
	Hepatopathy	13/420(3.1; 1.4–4.8)	3/325(0.9; -0.1–2.0)	10/95(10.5; 4.4–16.7)
	Chronic bronchitis	12/420(2.9; 1.3–4.5)	1/325(0.3; -0.3–0.9)	11/95(11.6; 5.1–18)
	Hyperuricemia	7/420(1.7; 0.4–2.9)	7/325(2.2; 0.6–3.7)	–
	Malignant tumor	6/420(1.4; 0.3–2.6)	3/325(0.9; -0.1–2.0)	3/95(3.2; -0.4–6.7)
	Chronic nephritis	4/420(1.0; 0.0–1.9)	1/325(0.3; -0.3–0.9)	3/95(3.2; -0.4–6.7)
	Cerebral apoplexy	4/420(1.0; 0.0–1.9)	1/325(0.3; -0.3–0.9)	3/95(3.2; 0.4–6.7)
**Smoking status-no./total no. (%; 95% CI)**				
	Yes	44/420(10.5; 7.5–13.4)	35/325(10.8; 7.4–14.1)	9/95(9.5; 3.6–15.4)
	No	376/420(89.5; 86.6–92.5)	290/325(89.2; 85.9–92.6)	86/95(90.5; 84.6–96.4)
**Alcohol consumption-no./total no. (%; 95% CI)**				
	Yes	44/420(10.5; 7.5–13.4)	32/325(9.8; 6.6–13.1)	12/95(12.6; 6.0–19.3)
	No	376/420(89.5; 86.6–92.5)	293/325(90.2; 86.9–93.4)	83/95(87.4)

### Application of Comprehensive Intervention

Comprehensive intervention included Baduanjin exercise, Chinese herbal medicine, moxibustion with acupoint application, and foot baths. Baduanjin exercise (100%) and Chinese herbal medicine prescriptions (90.5%) were most widely used (**Table 2**). The frequency of patients receiving various treatment combinations in the intervention group was shown in the supplementary materials (**Table S1**).

**Table 2:** Comprehensive intervention and Positive RT-PCR test.

		All patients	Comprehensive intervention	No intervention
**Comprehensive intervention-no./total no. (%; 95% CI)**				
	Baduanjin exercise		325/325(100)	
	Tongzhi granule	–	294/325(90.5; 87.3–93.7)	–
	Wuhan Kangyi decoction	–	39/325(12.0; 8.5–15.5)	–
	Moxibustion with acupoint application	–	90/325(27.7; 22.8–32.6)	–
	Foot bath	–	19/325(5.8; 3.3–8.4)	–
**Positive RT-PCR test-no./total no.(%; 95% CI)**		24/420(5.7; 3.5–7.9)	9/325(2.8; 1.0–4.6)	15/95(15.8; 8.5–23.1)

### Symptoms Before RT-PCR Testing

57.4% (241/420; 95% CI: 52.7–62.1%) subjects still manifested symptoms at the end of the observation period, 22.6% (95/420; 95% CI: 18.6–26.6%) of them showed insomnia, other common symptoms included cough [22.4%(94/420; 95% CI: 18.4–26.4%)], shortness of breath [21.0% (88/420; 95% CI: 17.1–24.8%)] and sweating [16.4% (69/420; 95% CI: 12.9–20%)] (**Table 1**). Besides, there was a difference between no intervention and comprehensive intervention in proportion of subjects accompanied symptoms (53.2 vs. 71.6%).

### RT-PCR Test Results

At the end of the observation period, 420 subjects had completed at least one RT-PCR test. It was found that the overall recurrent rate of positive RT-PCR test results was 5.7% (24/420; 95% CI: 3.5–7.9%), 2.8% (9/325; 95% CI: 1.0–4.6%) in the comprehensive intervention group, and 15.8% (15/95; 8.5–23.1%) in the non-intervention group (**Table 2**).

### Analysis of Positive RT-PCR Test Results

We performed a logistic regression analysis using factors including comprehensive intervention (yes/no), age, sex, BMI, course of disease, symptom, comorbidity, smoking status, and alcohol consumption to analyze the relations of positive RT-PCR test results. The results of univariate analysis showed that age, comorbidity, and intervention of the observed subjects were related to positive RT-PCR test results (*P* < 0.05). Multivariate analysis revealed that intervention was related to positive RT-PCR test results (*P* < 0.05), suggesting that comprehensive intervention method might be a protective factor for positive RT-PCR test results (**Table 3**). Detailed logistic regression analysis was shown in the supplementary materials (**Tables S2**-**S4**).

**Table 3:** Univariate Analysis and Multivariate Analyses for the Positive RT-PCR test.

	Univariate Analyses		Multivariate analyses	
	P	OR (95% CI)	P	OR (95% CI)
Management (comprehensive intervention vs. no intervention)	<0.001	0.152 (0.064, 0.360)	<0.001^1^	0.169 (0.070, 0.408)
			<0.001^2^	0.169 (0.070, 0.408)
			<0.001^3^	0.162 (0.066, 0.395)
			<0.001^4^	0.169 (0.069, 0.412)
			<0.001^5^	0.166 (0.067, 0.407)
			<0.001^6^	0.165 (0.067, 0.406)
Sex (M vs. F)	0.623	1.231 (0.538, 2.813)	0.448	1.394 (0.592, 3.286)
			0.451	1.395 (0.587, 3.318)
			0.170	1.891 (0.760, 4.705)
			0.198	1.825 (0.730, 4.564)
			0.207	1.806 (0.721, 4.525)
			0.210	1.800 (0.718, 4.510)
Age	0.026	1.040 (1.005, 1.076)	0.127	1.026 (0.993, 1.062)
			0.129	1.026 (0.992, 1.062)
			0.093	1.030 (0.995, 1.065)
			0.189	1.024 (0.988, 1.062)
			0.184	1.025 (0.989, 1.062)
			0.183	1.025 (0.989, 1.062)
BMI	0.999	1.000 (0.890, 1.123)	0.988	0.999 (0.893, 1.118)
			0.913	0.994 (0.888, 1.112)
			0.755	0.981 (0.871, 1.105)
			0.716	0.978 (0.869, 1.102)
			0.704	0.977 (0.868, 1.101)
Smoking Status (Y vs N)	0.998	NA^*^	0.997	NA
			0.997	NA
			0.997	NA
			0.997	NA
Alcohol Use (Y vs. N)	0.725	0.766 (0.174, 3.374)	0.951	0.951 (0.188, 4.804)
			0.998	0.998 (0.197, 5.051)
			0.958	1.045 (0.206, 5.309)
			0.946	1.058 (0.208, 5.373)

*Estimators were not available because one cell contained zero value.

^1^Model included site, age and sex.

^2^Model included site, age, sex and BMI.

^3^Model included site, age, sex, BMI, smoking status and alcohol use.

^4^Model included site, age, sex, BMI, smoking status, alcohol use and disease history.

^5^Model included site, age, sex, BMI, smoking status, alcohol use, disease history and symptoms status when entering isolation site.

^6^Model included site, age, sex, BMI, smoking status, alcohol use, disease history, symptoms status when entering isolation site, and the duration from initial symptoms onset to nucleic retest.

## Discussion

On February 25, 2020, the Guangdong Provincial CDC released preliminary statistics, which showed that the proportion of cured patients presenting with positive RT-PCR test results discharged in Guangdong Province was approximately 14%. Another study reported that after four COVID-19 patients (all medical personnel) were cured, RT-PCR test result from pharyngeal swabs appeared positive again (Lan et al., 2020). The Eighth People’s Hospital of Guangzhou City also continued to follow up COVID-19 patients discharged from hospital, finding that the recurrent rate of positive RT-PCR test results was 9.6%. Repeated fluctuation-positive RT-PCR test results have drawn widespread attention globally. It’s unclear whether patients who meet the current clinical recovery criteria are completely cured. In the process of continuously exploring the disease, we have focused on whether symptoms recurred in the population, the time when the RT-PCR test result turned negative again, the infectivity of the population, the reason, significance, and related factors of positive RT-PCR results in cured COVID-19 patients.

The causes of positive RT-PCR test results in cured patients have been researched at present (Ling et al., 2020). Considering the biological characteristics of SARS-CoV-2 and reduced reinfection of cured patients, the positive RT-PCR test results are probably due to the presence of virus residues in the body. Moreover, negative RT-PCR test results cannot rule out the possibility of COVID-19 (National Health Commission of the People’s Republic of China, 2020b). There are some factors that may cause false negatives, which could lead to misjudgment of cure in patients. These potential factors include sensitivity of the RNA extraction kit and specimen collection process methods. The production of kits has faced increased demands due to a sudden epidemic situation, the research and development time is extremely limited, the process is simplified, and the quality of the kits is unstable, all of which affect the sensitivity of the kits. Specimen collection method, collection time, storage and transportation also have a certain impact on the RT-PCR test result (Yang et al., 2020). For example, when sampling with swabs, if the sampling time is too short to collect the virus RNA, false negative results will occur. In addition, the application of medicine is also an important factor in the misjudgment of negative RT-PCR test results. The use of glucocorticoid and other medicines were found to negatively affect the body’s immune balance and inhibit inflammatory response, causing delays in eliminating the virus (Torres et al., 2015). According to the latest research, SARS-CoV-2 can detoxify for up to 37 days (Zhou et al., 2020). Viral pneumonia usually maintains a longer recovery period, including COVID-19. This characteristic manifestation of COVID-19 has increased the difficulty of objectively and precisely assessing the patient’s lung recovery using computed tomography (CT), and therefore affects accurate recovery judgment. In addition, recurrent positive RT-PCR test results are also associated with the patients’ autoimmune function system and comorbidity (Ling et al., 2020).

In response to the increasing number of reports of positive RT-PCR test results in recovered COVID-19 patients, the Center for Disease Prevention and Control has made an immediate response and adjustment, which is to strengthen the continuous investigation and detection of RT-PCR test during the quarantine of cured patients, and strengthen follow-up and health guidance. Experts suggest that if recurrent positive RT-PCR test results occur, a quick re-test in the short term will benefit to rule out the misjudgment caused by technology. RT-PCR tests of nasopharyngeal swabs combined with anal swabs also contribute to improving the accuracy of the assessment of viral status. In addition, scientists have proposed a layered discharge strategy for different types of patients, which reflects more individualized assessment methods consistent with clinical practice, by increasing the number of RT-PCR tests and the criteria for patients’ hospital discharge and quarantine to reduce positive conversion ratio. It is worth noting that antibody testing has been incorporated into the diagnostic standards of COVID-19, and new corresponding measures, including viral antibody testing, have gradually begun to introduced in discharged patients to promote objective assessment of patients’ recovery status.

In the course of clinical practice, we found that patients who received comprehensive intervention had fewer events of positive RT-PCR test results recurrence than patients who did not receive intervention. In this study, we explored the factors that influence the RT-PCR test results. By univariate logistic regression analysis, it was found that age, combined underlying diseases, and intervention methods were correlated with positive RT-PCR test results recurrence (P < 0.05). Previously published literature has reported that age, gender, and underlying diseases were risk factors for COVID-19 (Li et al., 2020; Wang et al., 2020; Zhou et al., 2020). Considering the potential influence of age, gender and underlying diseases on the nucleic acid reactivation results, which may interfere with the actual relationship between the intervention methods and PT-PCR results. Consequently, factors such as age, gender, and underlying diseases need to be corrected. After adjusting for these factors in multivariate analysis, we found the actual relationship between the intervention methods and positive RT-PCR test results recurrence. That was, the comprehensive intervention mode is the protective factors of positive RT-PCR test results recurrence. The comprehensive interventions in our study including Baduanjin exercise, foot baths, moxibustion with acupoint application and Chinese medicine may strengthen the immune system (Tong et al., 2020; Zou et al., 2018), restore the body’s metabolic balance, and promote elimination of residual viruses from the body; all these effects might reduce the proportion of positive RT-PCR test results in discharged COVID-19 patients.

Though positive RT-PCR test results and the ability to transmit the virus in patients who have been discharged, still remain unreasonable explanation. The comprehensive intervention therapy used in our study can reduce the occurrence of positive RT-PCR test, and its mechanism may be related to improving the body’s immune function, promoting the recovery of the body’s metabolic balance, and accelerating the excretion of residual viruses in the body. The comprehensive intervention therapy could be recommended for COVID-19 patients who have been discharged from hospital, and entered the rehabilitation stations for the 14 days period of clinical observation. The comprehensive intervention therapy can promote early recovery of patients, reduce the recurrence of positive RT-PCR test results and prevent secondary transmission, which provides a reference experience for the prevention and control of the recurrence phenomenon.

However, the specific targets and mechanisms of the intervention need to be further explored. Considering the small sample size of our study and the fact that the method of quantitative detection of viral antibody has not been adopted, the results of this study need further confirmation.

## Data Availability Statement

The raw data supporting the conclusions of this article will be made available by the authors, without undue reservation.

## Ethics Statement

The studies involving human participants were reviewed and approved by Medical Ethics Committee of Hubei Provincial Hospital of Traditional Chinese Medicine (no. HBZY2020-C01-01). Written informed consent for participation was not required for this study in accordance with the national legislation and the institutional requirements.

## Author Contributions

XT, FL, SH, XDL, and YiZ designed the study, took responsibility for the integrity of the data and the accuracy of the data analysis, and had final responsibility for the decision to submit for publication. MX, XM, XYL, QZ, JK, JuT, and YL contributed to data acquisition. YaZ analyzed the data. FL and YaZ contributed to data interpretation. YaZ, QL, JuT, FL, and YaZ contributed to data sorting and cleaning. FL, JiT, DJ, YuZ, QL, QD, ST, JL, PS, HW, ZJ, ZZ, and JS drafted the manuscript. All authors contributed to the article and approved the submitted version.

## Funding

This work was funded by the Special Project for Emergency of the Ministry of Science and Technology (2020YFC0845000) and the Traditional Chinese Medicine Special Project for COVID-19 Emergency of National Administration of Traditional Chinese Medicine (2020ZYLCYJ04-1).

## Conflict of Interest

The authors declare that the research was conducted in the absence of any commercial or financial relationships that could be construed as a potential conflict of interest.
