# Supplementary material for: Positive RT-PCR Test Results in 420 Patients Recovered From COVID-19 in Wuhan: An Observational Study
Source: Front Pharmacol. 2020 Oct 7;11:549117. doi: 10.3389/fphar.2020.549117 (PMC7577046; doi:10.3389/fphar.2020.549117)
Supplement: Supplementary file 1 [file DataSheet_1.docx]

**Supplementary materials**

**Table（S1）The frequency of patients receiving various treatment combinations in the intervention group**

|  | **Negative RT-PCR test in case (N=316)** | **Positive RT-PCR test in case**  **(N=9)** | **Intervention group**  **(N=325)** |
| --- | --- | --- | --- |
| Tongzhi Granule + Baduanjin exercise | 175 (55.4%) | 6 (66.7%) | 181 (55.7%) |
| Tongzhi Granule + Moxibustion with acupoint application + Baduanjin exercise | 68 (21.5%) | 2 (22.2%) | 70 (21.5%) |
| Wuhan Kangyi Decoction + Tongzhi Granule + Baduanjin exercise | 24 (7.6%) | 0 (0.0%) | 24 (7.4%) |
| Baduanjin exercise | 22 (7.0%) | 0 (0.0%) | 22 (6.8%) |
| Tongzhi Granule + Moxibustion with acupoint application + Foot bath + Baduanjin exercise | 10 (3.2%) | 1 (11.1%) | 11 (3.4%) |
| Wuhan Kangyi Decoction + Baduanjin exercise | 4 (1.3%) | 0 (0.0%) | 4 (1.2%) |
| Wuhan Kangyi Decoction + Tongzhi Granule + Moxibustion with acupoint application + Baduanjin exercise | 3 (0.9%) | 0 (0.0%) | 3 (0.9%) |
| Wuhan Kangyi Decoction + Tongzhi Granule + Moxibustion with acupoint application + Foot bath + Baduanjin exercise | 3 (0.9%) | 0 (0.0%) | 3 (0.9%) |
| Wuhan Kangyi Decoction + Moxibustion with acupoint application + Baduanjin exercise | 2 (0.6%) | 0 (0.0%) | 2 (0.6%) |
| Tongzhi Granule + Foot bath + Baduanjin exercise | 1 (0.3%) | 0 (0.0%) | 1 (0.3%) |
| Wuhan Kangyi Decoction + Tongzhi Granule + Foot bath + Baduanjin exercise | 1 (0.3%) | 0 (0.0%) | 1 (0.3%) |
| Wuhan Kangyi Decoction + Moxibustion with acupoint application + Foot bath + Baduanjin exercise | 1 (0.3%) | 0 (0.0%) | 1 (0.3%) |
| Wuhan Kangyi Decoction + Foot bath + Baduanjin exercise | 1 (0.3%) | 0 (0.0%) | 1 (0.3%) |
| Foot bath + Baduanjin exercise | 1 (0.3%) | 0 (0.0%) | 1 (0.3%) |
|  |  |  |  |

**Table（S2）Univariate Analysis and Multivariate Analyses for the Positive RT-PCR test**

| **Variable** | **Univariable OR (95%CI)** | **P-value** |
| --- | --- | --- |
| Wuhan Kangyi Decoction (Y vs N) | <0.001 (<0.001, >999.999) | 0.965 |
|  |  |  |
| Tongzhi Granule (Y vs N) | 0.234 (0.099, 0.549) | <.001 |
|  |  |  |
| Moxibustion with acupoint application (Y vs N) | 0.507 (0.148, 1.741) | 0.281 |
|  |  |  |
| Foot bath (Y vs N) | 0.913 (0.117, 7.144) | 0.931 |
|  |  |  |
| Baduanjin exercise (Y vs N) | 0.152 (0.064, 0.360) | <.001 |
|  |  |  |
| Sex (M vs. F) | 1.231 (0.538, 2.813) | 0.623 |
|  |  |  |
| Age | 1.040 (1.005, 1.076) | 0.026 |
|  |  |  |
| BMI | 1.000 (0.890, 1.123) | 1.000 |
|  |  |  |
| Smoking Status（Y vs N） |  | 0.962 |
|  |  |  |
| Alcohol use (Y vs N) | 0.766 (0.174, 3.375) | 0.725 |
|  |  |  |

**Table（S3）Univariate Analysis and Multivariate Analyses for the Positive RT-PCR test in Tongzhi Granule**

| **Variable** | **P-value Multivariate OR (95%CI)^[1]^** | **P-value Multivariate OR (95%CI)^[2]^** | **P-value Multivariate OR (95%CI)^[3]^** | **P-value Multivariate OR (95%CI)^[4]^** | **P-value Multivariate OR (95%CI)^[5]^** | **P-value Multivariate OR (95%CI)^[6]^** |
| --- | --- | --- | --- | --- | --- | --- |
| Tongzhi Granule | 0.001 0.236 (0.099, 0.561) | 0.001 0.236 (0.099, 0.561) | 0.001 0.230 (0.095, 0.552) | 0.001 0.237 (0.098, 0.574) | 0.001 0.235 (0.097, 0.569) | 0.001 0.232 (0.096, 0.565) |
|  |  |  |  |  |  |  |
| Sex (M vs. F) | 0.399 1.443 (0.616, 3.380) | 0.402 1.444 (0.612, 3.406) | 0.169 1.883 (0.765, 4.637) | 0.199 1.811 (0.731, 4.486) | 0.207 1.794 (0.723, 4.452) | 0.207 1.795 (0.724, 4.453) |
|  |  |  |  |  |  |  |
| Age | 0.038 1.035 (1.002, 1.070) | 0.038 1.035 (1.002, 1.070) | 0.027 1.038 (1.004, 1.073) | 0.088 1.031 (0.995, 1.069) | 0.084 1.032 (0.996, 1.069) | 0.084 1.032 (0.996, 1.069) |
|  |  |  |  |  |  |  |
| BMI |  | 0.992 0.999 (0.894, 1.117) | 0.940 0.996 (0.890, 1.114) | 0.772 0.983 (0.872, 1.107) | 0.744 0.980 (0.871, 1.104) | 0.731 0.980 (0.871, 1.102) |
|  |  |  |  |  |  |  |
| Smoking Status |  |  | 0.970 <0.001 (<0.001, >999.999) | 0.970 <0.001 (<0.001, >999.999) | 0.970 <0.001 (<0.001, >999.999) | 0.970 <0.001 (<0.001, >999.999) |
|  |  |  |  |  |  |  |
| Alcohol use |  |  | 0.977 1.024 (0.204, 5.132) | 0.940 1.064 (0.212, 5.350) | 0.911 1.097 (0.217, 5.538) | 0.899 1.111 (0.220, 5.598) |
|  |  |  |  |  |  |  |
| Disease history |  |  |  | 0.310 1.635 (0.633, 4.226) | 0.291 1.672 (0.644, 4.340) | 0.292 1.670 (0.644, 4.329) |
|  |  |  |  |  |  |  |
| Symptoms status when entering isolation site |  |  |  |  | 0.593 0.787 (0.326, 1.899) | 0.587 0.783 (0.324, 1.891) |
|  |  |  |  |  |  |  |
| Duration |  |  |  |  |  | 0.775 1.007 (0.958, 1.060) |
|  |  |  |  |  |  |  |

| * Estimators were not available because one cell contained zero value. |
| --- |
| 1. Model included Tongzhi granule, age and sex |
| 2) Model included Tongzhi granule, age, sex and BMI. |
| 3) Model included Tongzhi granule, age, sex, BMI, smoking status and alcohol use. |
| 4) Model included Tongzhi granule, age, sex, BMI, smoking status, alcohol use and disease history. |
| 5) Model included Tongzhi granule, age, sex, BMI, smoking status, alcohol use, disease history and symptoms status when entering isolation site. |
| 6) Model included Tongzhi granule, age, sex, BMI, smoking status, alcohol use, disease history, symptoms status when entering isolation site, and the duration. |

**Table（S4）Univariate Analysis and Multivariate Analyses for the Positive RT-PCR test in Baduanjin exercise**

| **Variable** | **P-value Multivariate OR (95%CI)^[1]^** | **P-value Multivariate OR (95%CI)^[2]^** | **P-value Multivariate OR (95%CI)^[3]^** | **P-value Multivariate OR (95%CI)^[4]^** | **P-value Multivariate OR (95%CI)^[5]^** | **P-value Multivariate OR (95%CI)^[6]^** |
| --- | --- | --- | --- | --- | --- | --- |
| Baduanjin exercise | <.001 0.169 (0.070, 0.408) | <.001 0.169 (0.070, 0.408) | <.001 0.162 (0.066, 0.395) | <.001 0.169 (0.069, 0.412) | <.001 0.166 (0.067, 0.407) | <.001 0.165 (0.067, 0.406) |
|  |  |  |  |  |  |  |
| Sex (M vs. F) | 0.448 1.394 (0.592, 3.286) | 0.451 1.395 (0.587, 3.318) | 0.170 1.891 (0.760, 4.705) | 0.198 1.825 (0.730, 4.564) | 0.207 1.806 (0.721, 4.525) | 0.210 1.800 (0.718, 4.511) |
|  |  |  |  |  |  |  |
| Age | 0.127 1.026 (0.993, 1.062) | 0.129 1.026 (0.992, 1.062) | 0.093 1.030 (0.995, 1.065) | 0.189 1.024 (0.988, 1.062) | 0.184 1.025 (0.989, 1.062) | 0.183 1.025 (0.989, 1.062) |
|  |  |  |  |  |  |  |
| BMI |  | 0.988 0.999 (0.893, 1.118) | 0.913 0.994 (0.888, 1.112) | 0.755 0.981 (0.871, 1.105) | 0.716 0.978 (0.869, 1.102) | 0.704 0.977 (0.868, 1.101) |
|  |  |  |  |  |  |  |
| Smoking Status |  |  | 0.967 <0.001 (<0.001, >999.999) | 0.967 <0.001 (<0.001, >999.999) | 0.967 <0.001 (<0.001, >999.999) | 0.967 <0.001 (<0.001, >999.999) |
|  |  |  |  |  |  |  |
| Alcohol Use |  |  | 0.951 0.951 (0.188, 4.804) | 0.998 0.998 (0.197, 5.051) | 0.958 1.045 (0.206, 5.310) | 0.946 1.058 (0.208, 5.373) |
|  |  |  |  |  |  |  |
| Disease history |  |  |  | 0.358 1.565 (0.603, 4.061) | 0.336 1.601 (0.614, 4.173) | 0.334 1.603 (0.616, 4.174) |
|  |  |  |  |  |  |  |
| Symptoms status when entering isolation site |  |  |  |  | 0.569 0.771 (0.315, 1.887) | 0.564 0.768 (0.313, 1.881) |
|  |  |  |  |  |  |  |
| Duration |  |  |  |  |  | 0.827 1.006 (0.956, 1.058) |
|  |  |  |  |  |  |  |

|  |  |
| --- | --- |
| * Estimators were not available because one cell contained zero value. | |
| 1. Model included Baduanjin exercise, age and sex | |
| 2) Model included Baduanjin exercise, age, sex and BMI. | |
| 3) Model included Baduanjin exercise, age, sex, BMI, smoking status and alcohol use. | |
| 4) Model included Baduanjin exercise, age, sex, BMI, smoking status, alcohol use and disease history. | |
| 5) Model included Baduanjin exercise, age, sex, BMI, smoking status, alcohol use, disease history and symptoms status when entering isolation site. | |
| 6) Model included Baduanjin exercise age, sex, BMI, smoking status, alcohol use, disease history, symptoms status when entering isolation site, and the duration. | |
